# Host-associated microbes mitigate the negative impacts of aquatic pollution

**DOI:** 10.1128/msystems.00868-24

**Published:** 2024-08-29

**Authors:** Rachel E. Diner, Sarah M. Allard, Jack A. Gilbert

**Affiliations:** 1 Department of Biological Sciences, University of Memphis, Memphis, Tennessee, USA; 2 University of California, San Diego, Scripps Institution of Oceanography, La Jolla, California, USA; 3 Department of Pediatrics, University of California, San Diego, La Jolla, California, USA; University of Connecticut, Storrs, Connecticut, USA

**Keywords:** bioremediation, aquatic pollution, microbiomes, environmental stress, microbiology, systems biology, coastal resilience

## Abstract

Pollution can negatively impact aquatic ecosystems, aquaculture operations, and recreational water quality. Many aquatic microbes can sequester or degrade pollutants and have been utilized for bioremediation. While planktonic and benthic microbes are well-studied, host-associated microbes likely play an important role in mitigating the negative impacts of aquatic pollution and represent an unrealized source of microbial potential. For example, aquatic organisms that thrive in highly polluted environments or concentrate pollutants may have microbiomes adapted to these selective pressures. Understanding microbe–pollutant interactions in sensitive and valuable species could help protect human well-being and improve ecosystem resilience. Investigating these interactions using appropriate experimental systems and overcoming methodological challenges will present novel opportunities to protect and improve aquatic systems. In this perspective, we review examples of how microbes could mitigate negative impacts of aquatic pollution, outline target study systems, discuss challenges of advancing this field, and outline implications in the face of global changes.

## PERSPECTIVE

Aquatic pollution is a global problem that threatens human well-being and impairs marine and freshwater ecosystems. Human populations are increasing, and the majority of people reside alongside oceans, rivers, and lakes (1, 2). These water bodies support important human activities such as providing water, food, recreational opportunities, and transportation. They also host diverse ecosystems that are valued for their cultural importance, tourism, conservation value, and biodiversity. Notably, many natural products—including drugs that treat human disease—are derived from aquatic systems (3). However, coastal development, urban runoff, industrial and agricultural operations, and a broad range of other human activities cause pollutants and excess nutrients to enter these aquatic systems. Naturally occurring marine and freshwater algae can also produce a variety of potentially harmful toxins. These influences can negatively impact aquatic organisms and impair ecosystem services (4).

Beyond ecosystem consequences, pollutant discharge causes well-documented negative impacts on human health and economic productivity. Toxicants such as heavy metals and a variety of organic pollutants harm humans through direct contact, ingestion of contaminated food, or drinking contaminated water (5–7). While some toxicants cause only minor health effects and can be metabolized by humans, other persistent pollutants and heavy metals can be acutely or chronically toxic and even bioaccumulate (8–10). A recent study estimated pollution is responsible for approximately 9 million human deaths per year, corresponding to one in six deaths worldwide (11), with pollution strongly impacting low- and middle-income countries. Poor water quality can also cause economic harm in the form of tourism losses, decreased property values, and costs to purify drinking water (12). Economically important aquaculture and fisheries industries may be impaired, posing risks to food security and food safety. For example, commercial and recreational fishing industries were heavily impacted by the Deepwater Horizon oil spill in the Gulf of Mexico, incurring estimated losses of over 25,000 jobs, $2.3 billion in industry output, $1.2 billion in total value added or gross regional product, $700 million in labor income, $160 million in state and local tax revenues, and $160 million in federal tax revenues (13). Additionally, microplastics are consistently found in marine and freshwater food species, and while the direct human health and economic impacts are poorly understood, their ability to transport adsorbed toxic chemicals is an increasing area of concern for food safety (14, 15). To protect coastal ecosystems and human well-being, managing the effects of aquatic pollution is increasingly critical and requires novel approaches. While the response of both humans and environmental macroorganisms to pollutants is heavily studied (5–7, 16), the functional features of microbes that live on and in these organisms (i.e., their microbiomes) are poorly understood and may possess the potential to mitigate negative effects of aquatic pollutants. Microbes are immensely diverse and can degrade a variety of pollutants (17–20). Most microbes studied for these benefits are free-living or live in sediments and soil, in part because these systems are easier to study and are directly exposed to pollution. However, microbes with pollution mitigation potential may also be associated with aquatic plants and animals in the environment, representing an uncharacterized and unconsidered possibility to improve host, environmental, and/or human health. Understanding and developing the potential of these microbial communities are important to (i) understand the true and “holistic” value of current aquatic ecosystem services; (ii) discover new microbial potential for bioremediation applications; and (iii) optimize integrative biological systems for *in situ* bioremediation and ecosystem resilience, from the microbial through the ecosystem level.

This perspective builds in an interdisciplinary manner upon prior concepts (e.g., microbial bioremediation, aquatic organismal ecotoxicology) to elucidate the potential of host-associated microbes in aquatic systems and identify study systems and methodologies that would be ideal for harnessing potentially beneficial microbial functions.

### How host-associated microbes can mitigate the negative impacts of aquatic pollution

Across environments and biological systems, host-associated microbes play an important role in host and environmental health. Mechanistic microbial interactions with pollutants including degradation, tolerance, and sequestration likely occur in and on diverse host organisms. In fact, chronic exposure of host–microbe systems to pollutants may provide adaptive pressure toward acquiring and diversifying these mechanisms (21). There are two broad ways, in which host-associated microbes may mitigate the impacts of aquatic pollutants. First, host-associated microbes may transform toxin pollutants into less toxic forms or sequester them from the host and environment. Second, host-associated microbes that are critical for host health and are resistant or resilient to pollution will more effectively sustain these holobiont systems (i.e., hosts and their associated species that operate as an ecological unit) than those that are not.

The diversity, and thus the potential, of aquatic host-associated microbes is vast (22–24). Animals, plants, and algae have tissue-specific and/or spatially distinct microbiomes. Animals also discharge microbes and nutrients in feces and pseudofeces, which can inoculate and transform surrounding sediment systems. If these microbes can reduce the toxicity of pollutants by degrading or sequestering them, this could benefit host health and ecosystem quality. Host-associated microbial communities could prevent toxicity to predators in the food web that might bioaccumulate or biomagnify pollutants. Even if hosts themselves can degrade a specific pollutant, degradation by native microbial communities may be energetically favorable for the host–microbe system. Finally, these microbial processes could improve food safety and reduce risks to human health associated with pollution-contaminated food sources or drinking water.

Pollutants may harm microbes critical to host health, and microbes that can withstand or degrade pollutants can continue providing beneficial services. There may be selective pressure for host-associated microbes to acquire these functions, either at the gene, species, or community level. For example, multiple microbial taxa may acquire genes for degrading or resisting pollutants, with this functional redundancy providing a buffer to the host against microbial community disturbance. These putative services can be direct, where microbes break down food (25, 26) to provide host sustenance or are antagonistic against specific pathogens (27). They can also be indirect, as healthy microbiomes are thought to protect against pathogenic infection by inhibiting pathogen colonization (28, 29).

One example of these potentially beneficial functions is host-associated microbes that degrade oil or toxic oil constituents, including polycyclic aromatic hydrocarbons (PAHs). Oil from routine drilling efforts, accidental spill events, transportation, and natural sources, is a major pollutant contaminating aquatic systems. Individual bacterial isolates cultured from oysters can grow on crude oil as a sole carbon source (30), and genome sequences of oyster-associated bacteria suggest the potential to degrade PAHs (31). Bacteria isolated from mussels (32) and corals (33) in oil-contaminated regions of the Persian Gulf are also capable of oil degradation. In one study, a microbial consortium isolated from coral was used to degrade oil, which improved coral health upon oil exposure (34). Several studies have demonstrated that exposure to oil or PAHs can alter the microbiomes of a variety of aquatic animals, which could lead to dysbiosis of the native microbiome and disruption of this barrier defense against pathogenic infections (e.g., reference 35).

Microbes associated with aquatic animals can also help reduce coastal burdens of nutrient pollution. Excess nutrients (particularly nitrogen and phosphorus) frequently enter aquatic systems from agricultural and urban activities. Nutrient over-enrichment can cause excess algal growth, which can impair water quality, reduce oxygen, and cause acute toxicity if algae are toxin-producing species. This eutrophication is estimated to cause $1B of damage to European coastal regions and over $2B to U.S. freshwater systems annually (36). Microbes present in the gut and other regions of aquatic animals can convert inorganic nitrogen (e.g., NO_3_) into nitrogen-based gasses through denitrification, which can then exit the aquatic system. Expansive studies have revealed that oyster reefs represent significant denitrification hotspots, which can reduce aquatic nitrogen pollution (37, 38). Furthermore, filter- and deposit-feeding aquatic animals from marine and freshwater systems (including snails, mussels, and aquatic insects) can contribute to the substantial conversion of NO_3_ into NO, a different nitrogen compound emitted into the atmosphere (39). The specific microbes responsible for these processes and their ecosystem roles are still poorly understood, despite potentially large biogeochemical impacts.

Numerous other pollutants can alter aquatic microbiomes. Extensive research explores how plastics impact aquatic host and environmental microbiomes (40), including gut microbiome alterations (41), and the potential for plastics to transport aquatic pathogenic microbes (42). Other pollutants shown to alter aquatic microbiomes include, but are by no means limited to, synthetic hormones (43), antibiotics (44), heavy metals (45, 46), and herbicides and pesticides that frequently end up in aquatic systems (47–50). While most of these studies examine pollutant impacts on host-microbial ecology, microbial community changes may generate hypotheses for investigating mechanistic interactions among microbes, pollutants, and hosts in a variety of systems.

Several factors influence how aquatic microbe–pollutant interactions broadly impact hosts and ecosystems. The capacity of host organisms to absorb or adsorb pollutants is an important consideration, which is influenced by host physiology, lifestyle, pollutant types, environmental conditions, and other system-specific factors. For instance, plastics are often expelled back into the environment through host egestion (51), while heavy metals and organic pollutants often accumulate in host tissues (52, 53), potentially increasing microbial access. Filter-feeding lifestyles can further intensify uptake and microbial access; laboratory experiments have demonstrated that mangrove oysters can absorb >90% of added phenanthrene (a common PAH pollutant), even with concentrations far above typical ambient levels (54) ability of microbes to degrade these pollutants, to which they have access, either alone or as part of a community, is also important. Understanding these interactions will help elucidate their broader impacts, which is crucial for accurately quantifying ecosystem services and implementing ecosystem-scale bioremediation strategies.

### Discovering existing and future benefits of aquatic host-associated microbes using appropriate study systems

**FIG 1:**
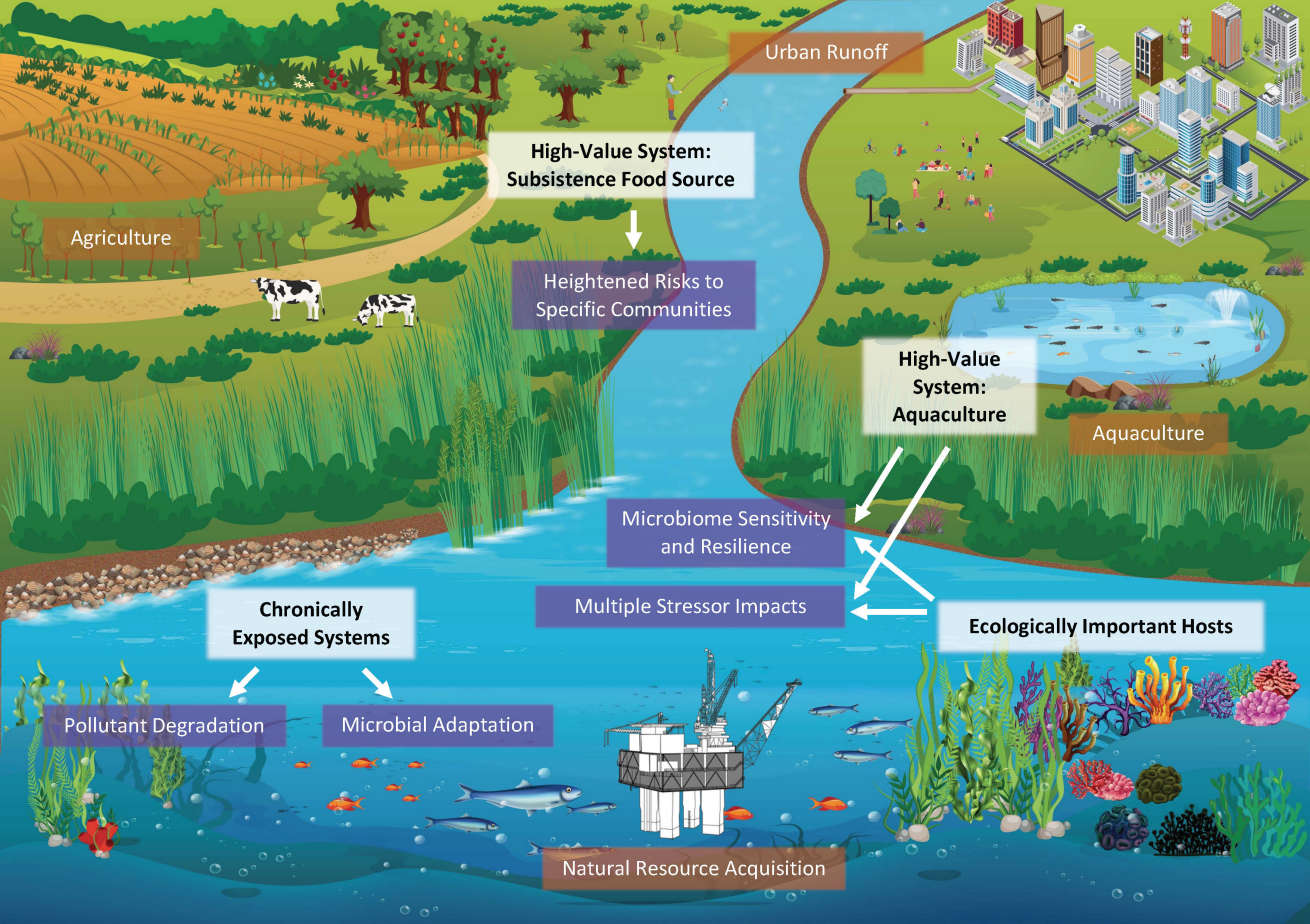
Pollution enters aquatic environments from many human sources (brown text boxes), including urban activities, agriculture, aquaculture, and natural resource acquisition activities. To understand and optimize how host-associated microbes mitigate the impacts of this pollution, focusing on particular study systems (white text boxes), and specific microbial processes relevant to these systems (purple text boxes) will be valuable. These include chronically exposed systems that are often unable to move away from pollution (e.g., oysters, mussels, and seagrass) and high-value systems from an economic or ecological perspective (e.g., aquaculture species or habitat-forming organisms such as coral reefs). Additionally, species harvested recreationally and for subsistence should be prioritized because these food sources are important and may pose heightened risks to particular communities.

The potential benefits discussed here can be systematically investigated in aquatic holobionts; however, it is critical to select the appropriate study system. Some especially relevant groups of experimental organisms include (i) high-value systems—macroorganism systems with high economic, conservation, cultural, or ecological value; (ii) chronically exposed systems—macroorganism systems that are highly likely to include beneficial microbes cause they are chronically exposed to pollutants that could drive microbial adaptations; and (iii) well-characterized systems—systems that, due to extensive prior research, are well-positioned for immediate potentially translational inquiries. In some cases, aims may overlap, pointing toward especially relevant model systems (Fig. 1).

#### High-value host–microbe systems

To promote conservation of specific threatened or ecologically important hosts, or to enhance the health of economically important organisms, specific species or close relatives should be used. Many important animals, including several aquaculture species, are not regularly exposed to pollutants. However, runoff events due to heavy rainfall or catastrophic events (e.g., oil spills or fires) can infrequently deliver detrimental pollution concentrations. Multiple environmental stressors, in conjunction with pollution, also threaten these organisms. Some examples include ecological keystone species, species that structurally support coastal ecosystems (e.g., corals and reef-building shellfish, seagrass), and fish and shellfish species prominent in aquaculture, recreational, and/or subsistence harvesting (e.g., oysters, mussels, catfish, tilapia, salmon). Several recent studies have demonstrated that pollutants can cause structural and in some cases even functional changes in these organisms’ microbiomes (46, 55–58). Beyond studying the impacts of pollution on these host–microbe systems generally, the impacts of acute pollution events should be examined in the context of microbiome resilience, standing genetic diversity of the microbiome, multiple environmental stressors, and interactions between the microbiome and host health.

#### Chronically exposed host–microbe systems

To characterize the potential of host-associated microbes for use *in situ* or in more traditional bioremediation efforts (e.g., bioaugmentation and biofilters), host–microbe systems chronically exposed to pollution would likely yield a high representation and diversity of useful microbial functions. These functions are likely energetically expensive for microbes to maintain in the absence of pollution, and chronic pollution provides selective pressure to acquire and retain them. This may be especially critical for complex and high-molecular-weight pollutant compounds that are challenging for even microbes to degrade, such as plastics and many persistent organic pollutants (59–61). Some ecological features that could increase microbial adaptations to pollution through chronic exposure include mobility (sessile organisms are unable to physically escape pollution), feeding mechanisms (filter-feeding organisms often concentrate pollutants from the environment), and in some cases feeding preferences (larger organisms that feed on toxin-accumulating smaller organisms). Examples of these organisms include corals, sponges, marine and freshwater bivalves, and other filter-feeding mollusks, seagrass, macroalgae, and coastal plants such as mangroves. Comparative field studies and “natural experiments” may be especially illuminating (22), as would examining similar host organisms in differentially polluted environments or different hosts in the same polluted environments.

#### Well-characterized host–microbe systems

Well-developed model systems are well situated for making discoveries that could apply to non-model systems. Traditional aquatic toxicology models such as zebrafish (*Danio rerio*) or crustaceans like *Daphnia magna* have been used to study impacts on the microbiome (e.g., 62–64). Several species of corals can be grown in the laboratory and studied for their responses to disease, environmental change, and pollution (65, 66). Additionally, extensive research has been conducted to characterize the microbiomes of seagrass, algae, shellfish, and many species of aquatic fish and mammals (24, 67–69). Organisms that can be grown in the lab, genetically manipulated, cultured axenically (an especially challenging problem for filter-feeding organisms), and that are themselves incapable of degrading pollutants may be of interest in these studies as well. Working with well-characterized microbial isolates from these host systems may also advance a mechanistic understanding of microbe–pollutant interactions. For example, readily culturable microbes found in the aquatic environment and within also aquatic microbiomes, including *Vibrio* spp., *Bacillus* spp., *Pseudomonas* spp., and *Pseudoalteromonas* spp., can degrade or transform a variety of pollutants (70–74).

### Challenges and areas for growth in studying host–microbe–pollution interactions

To understand how pollution shapes important species’ microbiomes, and to discover new biological potential in host-associated microbes, there are a variety of methodological challenges to overcome. Fortunately, new technologies for working with whole microbial communities and microbial isolates promise potential progress for these fields.

Emerging experimental and computational approaches used in other study systems can be applied to this field. Multi-omics approaches can characterize host-associated microbes and their response to pollutants and link microbiome diversity and functions to animal health. For example, dual RNA-sequencing profiles gene expression in hosts and their microbial communities simultaneously and is often used to characterize host–pathogen interactions during infections (75). When examining pollutant degradation, multiple degradation products and microbes involved in various degradation steps should be tracked and computational tools utilized to understand community-wide degradation networks. Engineering hosts and microbiomes can facilitate functional genomics approaches that could be applied to investigate interactions with pollutants. Advanced cell-sorting and visualization techniques may also help elucidate many aspects of these interactions, for example, identifying where in host organisms pollutants are concentrating (76) or examining microbial community heterogeneity on multiple scales (77). Finally, when possible, experiments should be designed to examine host and microbiome responses both separately and together (e.g., reference 78). Both observational and controlled experimental approaches should be applied to investigate these systems at multiple biological levels (e.g., gene, protein, metabolite, and organismal physiology) on various time scales to untangle the individual and interacting effects of pollutants on hosts and their microbiomes.

Pollutants should be investigated with co-occurring environmental stressors. Multiple stressor studies are important for understanding how organisms respond to the natural environment now and in the future. However, these studies rarely consider aquatic host microbiomes, except in the context of known pathogenic microbes or microbial community profiles. Pollutants may interact with other stressors to negatively affect hosts and their microbes, leading to dysbiosis and increased disease. Environmental changes could also enhance microbial pollutant degradation. For example, high temperatures can increase microbial growth, metabolism, and pollutant degradation (79). However, the extent and duration of these changes in the context of bioremediation are complex (80) and rarely studied in aquatic systems. Thus, future studies should investigate the breadth of potential impacts and how host-associated microbes respond mechanistically to pollutants and physiochemical variables.

Finally, increasing the culturable minority of aquatic host-associated microbes is a critical step in harnessing microbial potential. Despite huge advances in omics technology, the gold standard for studying microbial function is the ability to culture and genetically manipulate a representative laboratory isolate. This has been notoriously challenging for host-associated microbes as many are obligate symbionts or adapted to highly specific host microenvironments (e.g., anaerobic conditions, microbes co-dependent on other microbes). Aquatic environments are highly diverse and dynamic, making it difficult to pinpoint a given microbe’s optimal conditions. Few published studies have systematically attempted to optimize culturing of host-associated microbes from aquatic systems though great strides are being made in the field of microbiology generally (81). Optimizing conditions such as media type, temperature, nutrient concentration, or even pollutant addition (e.g., there are several species of obligate PAH-degrading bacteria) is necessary. Furthermore, employing microcosm systems such as EcoFabs (21) could enable investigations of host–microbe systems *in situ* across space and time. Finally, fungi and algae are common members of aquatic microbiomes with the potential to degrade pollutants but require vastly different culturing approaches. Culturing these microbes, many of which have only been identified via sequencing, will present opportunities for new discoveries about microbial functions and their role in host and environmental systems.

### Harnessing the potential of host-associated microbes for a better world

Understanding how host–microbe systems respond to pollution has many potential applications. While these range in feasibility, the first step is gaining a better understanding of these integrated biological systems. Beneficial microbes could be used in bioremediation efforts, to improve coastal restoration efforts such as living shorelines, and to improve health in threatened and sensitive aquatic species. Furthermore, microbial pollutant mitigation may be especially useful for growing aquaculture industries.

Addressing this research gap is especially important in the face of changing environments, where pollution is increasing at the same time physiochemical factors threaten to stress aquatic systems. Microbes drive biogeochemical cycles in aquatic systems and provide important ecosystem services that are often uncharacterized and thus, not quantified in terms of environmental management and preservation. Several environmental alterations related to climate change can influence host microbial ecology (82–84) and bioremediation processes (85). However, it is not known whether these complex co-occurring variables will increase, decrease, or cause no impact on pollutant mitigation by aquatic microbes. Research efforts should also emphasize species harvested recreationally and for subsistence because these food sources are important and may pose heightened risks to particular communities (86). Ultimately, systematically pursuing a deeper understanding of these systems may reveal opportunities to mitigate the harm increasingly being caused by pollution in aquatic systems.

### Conclusion

The exploration of host-associated microbes in aquatic systems offers a promising avenue for mitigating the adverse effects of pollution. By harnessing these microbes’ natural abilities to degrade or sequester pollutants, we can potentially improve the resilience of aquatic ecosystems, enhance aquaculture productivity, and protect human health. However, realizing this potential requires overcoming significant scientific and technical challenges, including understanding the complex interactions among hosts, microbes, and pollutants, and developing effective strategies to leverage these interactions for bioremediation and conservation efforts. As we navigate these challenges, the interdisciplinary nature of this research underscores the importance of collaboration across fields to unlock the full potential of host-associated microbes in addressing one of the most pressing environmental issues of our time.
